# Effect of *Vitex agnus-castus *ethanolic extract on hypothalamic *KISS-1* gene expression in a rat model of polycystic ovary syndrome 

**Published:** 2021

**Authors:** Zoleykha Feyzollahi, Homa Mohseni Kouchesfehani, Hanieh Jalali, Delaram Eslimi-Esfahani, Abbas Sheikh Hosseini

**Affiliations:** 1 *Department of Animal Biology, Faculty of Biological Sciences, Kharazmi University, Tehran, Iran*

**Keywords:** Kisspeptins, Hypothalamus, Polycystic ovarian syndrome, Vitex agnuscastus extract, Ovarian follicles

## Abstract

**Objective:**

Polycystic ovary syndrome (PCOS) is an endocrine system disruption that affects 6-10% of women. Some studies have reported the effect of *Vitex agnus-castus* (Vitagnus) on the hypothalamic-pituitary-gonad axis (HPG). This study was conducted to investigate Vitagnus effect on the expression of kisspeptin gene in a rat model of PCOS.

**Materials and Methods:**

Thirty-two female rats were distributed into: control, Vitagnus-treatment (365 mg/kg for 30 days), PCOS (Letrozole for 28 days) and PCOS animals treated with Vitagnus (30 days of Vitagnus after PCOS induction). At the end of the treatments, serum and ovaries were collected for analysis. Expression level of *KISS-1* gene in the hypothalamus was investigated, using Real-Time-PCR.

**Results:**

In the PCOS group compared to control, FSH, progesterone and estradiol levels were decreased, whereas testosterone and LH levels were significantly increased. No significant changes were observed in the Vitagnus-treated animals in compare to control. However, Vitagnus treatment in the PCOS group, resulted in a raise in progesterone, estrogen and FSH levels and a reduction in the levels of testosterone and LH. Quantitative gene expression analysis showed that PCOS induction resulted in over-expression of *KISS-1* gene, however, Vitagnus treatment reduced this up-regulated expression to normal level.

**Conclusion:**

In conclusion, our results indicated that Vitagnus extract inhibited downregulation of *KISS-1* gene in the hypothalamus of PCOS rats. Because of the master role of kisspeptin in adjusting the HPG axis, Vitagnus is likely to show beneficial effects in the treatment of PCOS via regulation of kisspeptin expression. This finding indicates a new aspect of Vitagnus effect and may be considered in its clinical applications.

## Introduction

Polycystic Ovary Syndrome (PCOS) is a complex endocrine disorder that influences about 6 to 10% of women ofreproductive age (Mccartney Ch and Marshall, 2016 ▶). Women with this syndrome suffer from acne, obesity, hair loss, hypertension and irregular ovulation (Hoeger et al., 2014 ▶). Levels of endocrine hormones are altered in these patients and they are at risk of infertility as well as metabolic disorders, such as type 2 diabetes (Lizneva et al., 2016 ▶). Several therapeutic approaches have been proposed for PCOS, including changing lifestyle, surgery and medications (Badawy and Elnashar, 2011 ▶). Currently, the most popular therapeutic approach is administration of medications, such as clomiphenecitrate, metformin, tamoxifen (Radosh, 2009 ▶). In case of mild (a condition in which the symptoms of the disease are not severe) syndrome, treatment includes administration of oral contraceptives or traditional herbal remedies (Van Die et al., 2013 ▶). *Vitex agnus-castus* (Vitagnus) is one of the oldest herbal remedies used to treat menstrual disorders and female infertility (Van Die et al., 2013 ▶; Russo and Galletti, 1995 ▶).Some clinical trials have demonstrated the benefits of Vitagnus in the treatment of premenstrual and hyperprolactinaemia disorders (Van Die et al., 2013 ▶). *Vitex agnus-castus*extract contains dopamine, opioid and estrogen receptor’s ligands, which modulate the level of hormones and lower the level of prolactin (Chen et al., 2011 ▶). It regulates sex hormone levels, mainly through regulation ofthe pituitary gland and LH (luteinizing hormone) production (Heskes et al., 2018 ▶). Kisspeptin, a neuropeptide secreted from the hypothalamus, functions throughbinding and activation of G protein-coupled receptor, GPR54 (Trevisan et al., 2018 ▶). Kisspeptin/GPR54 system acts upstream of GnRH and controls various aspects of female fertility, such as puberty, ovulation and lactation, through negative and positive feedback systems of sex hormones (Tena-Sempere, 2006 ▶). In the hypothalamus of rodents, kisspeptin neurons are situated in the nuclei of arcuate (ARC) and anteroventral periventricular (AVPV) (Gottsch, 2004 ▶). In the ARC, kisspeptin neurons contribute to the production of GnRH/LH pulse through a negative feedback, whereas in the AVPV, kisspeptin neurons are under estrogen positive feedback control and are concerned with pre-ovulatory GnRH/LH surge generation (Kauffman et al., 2007a ▶). Studies performed in animal models have demonstrated that the expression level of hypothalamic *KISS*-1 mRNA is altered in reproductive disorders, such as PCOS (Witchel and Tena-Sempere, 2013 ▶). Manipulation of kisspeptin signaling may be a suitable curative strategy for the treatment of PCOS-related fertility disorders, so that medications that regulate this signaling system may be taken into consideration for PCOS treatment (Romero-Ruiz et al., 2019 ▶). Although several studies have been performed to investigate the effects of Vitagnus on PCOS, most of them focused on identifying hormonal and tissue changes, and few studies have been performed to identify genes affected by Vitagnus. Given the importance ofunderstanding the action of herbal medicines at genes level, in the present study, we investigated the effect of *Vitex agnus-castus *fruit ethanolic extract on the expression of hypothalamic *KISS-1* gene in a rat model of PCOS; Our assumption was that Vitagnus has a positive effect on regulating the levels of sex hormones to relieve issues associated with related to PCOS by regulating kisspeptin neurons activity.

## Materials and Methods

**Preparing** ***V.agnus-castus*** **fruit extract**

*Vitex agnus-castus *fruit was prepared from the School of Traditional Medicine, Shahid Beheshti University of Iran and its extract was prepared according to previous protocols (Liu, 2008 ▶). The fruits were dried understandard conditions, avoiding microbial contamination with a suitable ventilation. For extraction, 500 g of fruit powder was dissolved and kept in 1 l of 70% ethanol for 48 hours. Then, the contents of the container were strained and the collected solution was moved to a balloon. The solvent was eliminated using a rotary device set at 70°C.

**Animal**

Thirty-two adult female Wistar rats weighing 200-250 g, were purchased from the animal house of Tehran University (Tehran, Iran) and kept in a controlled environment of 12 hr light/dark cycle, 60-65% humidity and temperature 22±2°C, without any water and food (Behparvar Co. Iran) restriction. All procedures were performed regarding the instruction of animal care and ethical committee of Kharazmi University.

Animals were monitored for 14 days and those with at least two regular estrous cycles, were divided into four groups (n=8 for each group) as follows: Control (intact);Vitagnus group (sham) that received 365 mg/kg Vitagnusextract orally for 30 days (Nasri et al., 2007);PCOS group that received letrozole (Aburaihan Pharmacy Company, Iran) 1mg/kg orally for 28 days (Kakadia et al., 2019 ▶); and PCT group (PCOS animals treated with Vitagnus extract at the dose of 365 mg/kg for 30 days). In the PCT group, at the first step, letrozole was injected for 28 days to develop PCOS; after this stage where PCOS wasinduced, letrozole treatment was stopped, and Vitagnus was started and continued for 30 days at the dose of 365 mg/kg. 

**Serum collection and ELISA assay**

At the end of 30 days of treatments, animals were anesthetized using chloroform in a special containerand 3.5 ml of blood was drawn from their hearts. Blood serum was obtained by centrifugation at 3000 rpm and kept at-80°C. Serum concentrations of LH, FSH, progesterone, estrogen and testosterone were measured by ELISA based on them anufacturer’s instruction (Monobind Inc. USA).

**Morphological/morphometric analysis of ovaries**

Eight ovaries of each group were removed and fixed in Bouin’s solution, embedded in paraffin, sectioned by rotary microtome (5-µm thickness) and stained by Hematoxylin-Eosin. Morphometric analysis of ovaries and follicular counting were performed using a light microscope (Zeiss, Germany) at magnification of X400. In each ovary, different follicles including primordial, primary, pre-antral, antral, graafian cystic, and corpus luteum were counted. The thickness of the granulosa and theca layers was measured in all groups using eyepiece micrometer.

**Quantitative real-time PCR**

A quantitative Real-time PCR (qRT-PCR) assay was followed in an Eppendorf Master cycler EP Real Plex. Briefly, hypothalamus tissues were collected from four groups and kept at-80°C until the time of RNA extraction. RNA was extracted using a specific kit (ParsTous, Iran) and then, converted to cDNA using cDNA synthesis kit (ParsTous, Iran) by mixing the template RNA and kit components (Buffer mix, Enzyme mix andDiethyl pyrocarbonate water) in RNase-free tubesand incubated for 10 min at 25°C, then, incubated at 50°C for 60 min. All procedures were conducted as instructed by the manufacturer. The reaction was stopped by heating at 70°C for 10 min. Primers for rat *KISS-1* and *GAPDH*, as the housekeeping gene, were designed using Primer-BLAST - NCBI. The primer pairs were as follows: *KISS-1* forward: TGCTGCTTCTCCTCTGTGTG and reverse: GTTCCTGGGGTCCTGACTGTTG; *GAPDH* forward: AGTGCCAGCCT-CGTCTCATA and reverse: GATGGTGATGGGTTTCCCGT. To perform real-time PCR, SYBR Green Real Master Mix Kit was used according to the manufacturer’s directions (Takara, Japan). Briefly, DNA was denatured at 95°C for 2 min followed by 40 cycles of 30 sec at 95°C and 30 sec at 60°C. The relative expression of *KISS-1*was normalized to *GAPDH* and relative changes in gene expression were determined by the 2^_ΔΔCT^ method.

**Statistical analysis**

All experiments were performed at least three times. Data werestatistically analyzed using SPSS. 22 software. A pvalue<0.05 was considered significant.

## Results

**Morphological characteristicsof ovaries in PCOS and treatment groups**

**Figure 1:**
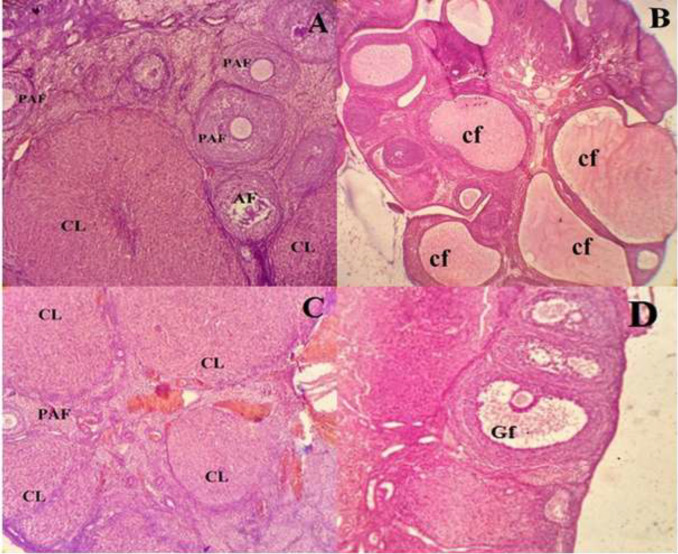
Histologic analysis of ovaries inletrozoletreated animals (PCOS animals) in comparison with healthy ovary and those treated with Vitagnus extracts. Ovarian sections stained with hematoxylin-eosin.A:Healthy ovary. B: Polycystic ovary has a large number of large cystic follicles with a thin granulosa and thick follicular sheath. C and D:Vitagnus-treated ovary with large follicles, thin follicular sheath and several corpus luteum. There was no significant difference in tissue characteristics between the control and Vitagnus groups. CL:corpus luteum, Cf:cystic follicles, AF:antral follicle, PAF:Preantral follicle, and Gf:Graafian follicle (x400).

Histological observations of ovaries in PCOS animals indicated that ovulation was impaired due to PCOS. Compared to the control animals, the number of large cystic follicles as well as small follicles washigher in ovaries of PCOS animals in compare to controls.Moreover, no corpus luteum was detected in PCOS animals and granulosa layers were thinner compared to that ofthe control group. Morphological studies showed that the number of cysts and their size were decreased in the PCOS rats treated with Vitagnus extract compared to the untreated PCOS animals. In the sham group in which animals were treated with Vitagnus extract for a period of 30 consecutive days, there was no change in the structure of ovary in comparison to the control group (Figure 1).

**Follicle count and measurement of theca and granulosa layers**

Counting different types of follicles in ovaries of the PCOS group indicated the presence of a large number of small follicles and abundance of large cystic follicles, which were characteristics of PCOS. The number of antral and Graafian follicles was significantly reduced in the PCOS group compared to the control ovaries. 

**Figure 2:**
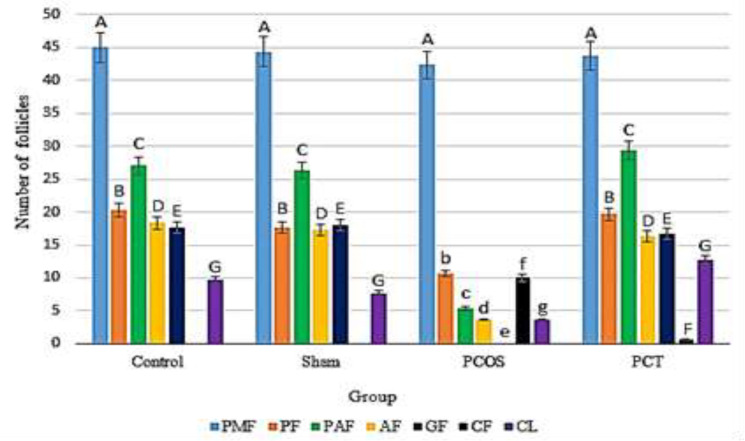
Count of different follicular types in the ovaries of control (non-treated animals), sham (Vitagnus treated animals), PCOS (letrozole treated animal) and PCT (letrozole and Vitagnus treated animals) groups. Different letters indicate a significant difference at p-value<0.05. PMF:primitive follicles;PF:Primary follicles;PAF:Preantral follicles;AF:Antral follicles;GF:graaf follicles;CF:Cystic Follicle and CL:Corpus luteum

**Figure 3:**
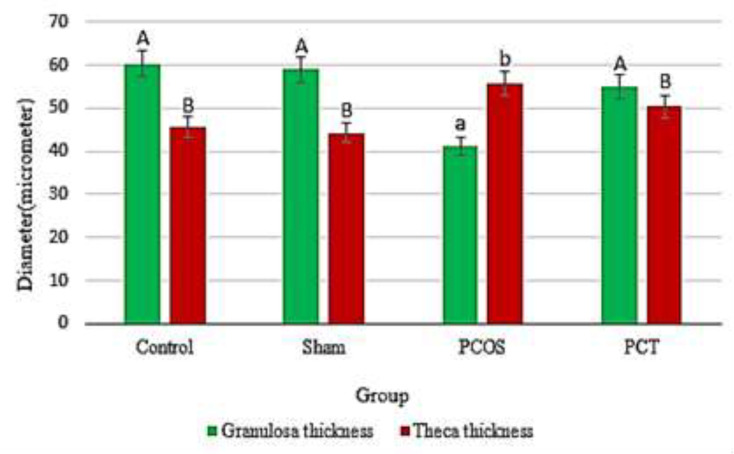
Thickness of the theca and granulosa layer in the ovaries of the control (non-treated animals), sham (Vitagnus treated animals), PCOS (letrozole treated animal) and PCT (letrozole and Vitagnus treated animals) groups. Different letters indicate a significant difference at p<0.05

The results of this study indicated that treatment of polycystic ovaries with Vitagnus extract led to reduced number and size of the cysts. Furthermore, a number of corpora lutea was found in most of the Vitagnus-treated PCOS ovaries, indicating the restoration of ovulation (Figure 2). Results of measuring the thickness of the theca and the granulosa layers of all groups indicated that there were no significant differences between the control and sham animals, however, theca layer was thicker butgranulosa layer was thinner in the PCOS ovaries compared to both the control and sham treated groups. In the PCT group, Vitagnus treatment reduced the thickness of theca layer butincreased that of granulosa layer in the ovaries (Figure 3).

**Analysis of sex hormones**

**Figure 4:**
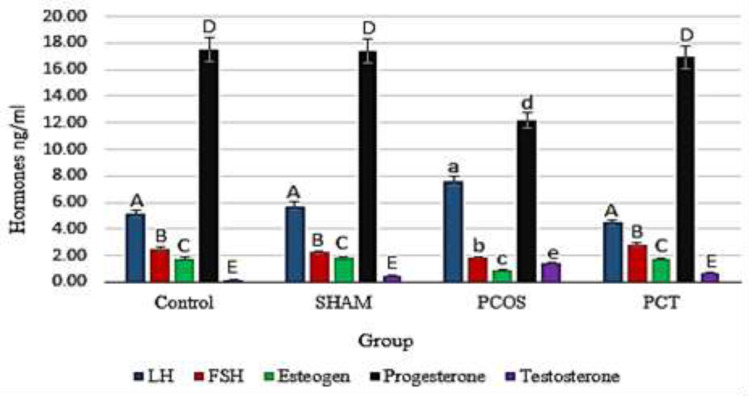
Levels of LH, FSH, testosterone, progesterone and estrogen hormones incontrol (non-treated animals), sham (Vitagnus treated animals), PCOS (letrozole treated animal) and PCT (letrozole and Vitagnus treated animals) groups. Different letters indicate a significant difference at p-value<0.05

The mean levels of LH, FSH, progesterone, estrogen and testosterone hormones in different groups are shown in Figure 4. *Vitex agnus-castus *extract, at 365 mg/kg, did not induce any significant changes in the levels of sex hormones in the sham group. Administration of letrozole to induce PCOS in rats, increased the levels of testosterone and LH, whereas it reduced progesterone, estrogen and FSH levels compared to the control group. Continuous treatment of PCOS animals with Vitagnus resulted in a significant reduction in testosterone and LH levels, however, FSH, estrogen and progesterone levels were significantly raised in these animals (Figure 4). 

***KISS-1*** **gene expression analysis**

**Figure 5:**
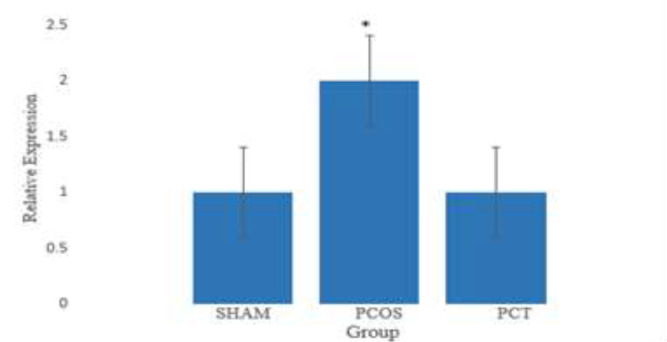
The relative gene expression of *KISS-1* in sham (Vitagnus treated animals), PCOS (letrozole treated animal) and PCT (letrozole and Vitagnus treated animals) groups compared to control group

The results of gene expression analysis revealed no significant differences in the expression of *KISS-1* gene between the control and Vitagnus-treated (sham) animals. Induction of PCOS with letrozole, led to a 2-fold increase in *Kiss-1* gene expression, where thedifference between control and PCOS groups was significant at p<0.05. Expression level of *Kiss-1* in the Vitagnus-treated PCOS animals (PCT) was comparable with the level of *Kiss-1* expression in the control animals and the difference was not significant (Figure5).

## Discussion

Consumption of herbal remedies by women has been increased over the past years and it was shown to be positively associated with a reduction of infertility issues (Arentz et al., 2014a ▶). Preclinical and clinical studies have investigated the effect of *Vitex agnus-castus *in the management of amenorrhea and abnormal ovulation (Kafali et al., 2004 ▶). There was an equivalent effect for bromocriptine and *Vitex agnus-castus* in PCOS women (Arentz et al., 2014b ▶).

In the present study, the effect of *Vitex agnus-castus *fruit on *KISS-1* gene expression was investigated in a rat model of letrozole-induced PCOS. Letrozole is a non-steroidal aromatase inhibitor and can greatly inhibit aromatase enzyme (Casper and Mitwally, 2011 ▶). Reduced activity of this enzyme inhibits aromatization of testosterone to estradiol, resulting in an increase in testosterone levels and a decrease in estradiol levels (Kauffman et al., 2007b ▶). The rat model of letrozole-induced PCOS was used in this study based on the low aromatase activity in PCOS women and considering the point that genetic variations in the aromatase gene (*CYP19*) are associated with the development of PCOS (Yang et al., 2018 ▶). Previous studies demonstrated that inducing PCOS with letrozole, led to the production of higher amounts of testosterone, elevated ratio of LH/FSH, highernumbers of follicular cysts, elevated thickness of the theca layer and decreased thickness of granulosa layer (Sun et al., 2013 ▶). All these key symptoms were observed in the current study, indicating successful induction of PCOS in our experimental animals. Treatment of the PCOS animals with Vitagnus extract led to a significant recovery of LH/FSH ratio to its normal level, a remarkable decline in testosterone level and a significant elevation in estrogen level. Moreover, Vitagnus treatment led to the presence of fewer follicular cyst, higher numbers of antral and Graafian follicles and reduced thickness of theca layer in PCOS animals. These results showed that Vitagnus extract relieved the symptoms associated with ovarian syndrome and led to the recovery of ovulation in the ovaries.

Aliabadi and colleagues counted kisspeptin neurons in the nuclei of arcuate (ARC) and AVPV of the hypothalamus in letrozole-induced PCOS rats. Their results revealed that the number of kisspeptin cells in the Arc nucleus increased under the letrozole influence, however, the number of kisspeptin neurons in AVPV nuclei wasdecreased (Aliabadi et al., 2017 ▶). Hypothalamic neurons of kisspeptin present estrogen receptor α (ERα) and were shown to be critically important sites for estrogen (E2)-mediated negative and positive feedback. E2 inhibits expression of kisspeptin in the ARC and stimulates it in the AVPV via ERα (Dubois et al., 2015 ▶). In addition, there is a temporary pairing between kisspeptin and LH release, which leads to positive correlations between kisspeptin and LH level in PCOS patients (Katulski et al., 2018 ▶). With regard to Aliabadi et al. findings, it seems that the ARC nucleus activity should be greater than that in the AVPV nucleus in the letrozole-induced PCOS. Therefore, due to the reduced level of estrogen, resulted from letrozole treatment, as well as the elevated level of LH, the expression of *KISS-1* is expected to be up-regulated (Kondo et al., 2016 ▶). The findings of the current study indicated that the level of LH was elevated whileestrogen level was reduced in the PCOS animals.Moreover, the level of *KISS-1* expression in the PCOS animals was significantly greater than that of healthy animals. These results are completely compatible with our hypothesis and those of the former studies that indicated higher activity of ARC nucleus and elevated expression levels of *KISS-1* in the letrozole-induced PCOS rats (Tang et al., 2019 ▶). Investigation of the effect of Vitagnus on PCOS animals revealed that treatment with Vitagnus not only reversed the level of sex hormones back to the normal state, but also maintained the expression level of *KISS-1*at its normal level. In other words, treatment with Vitagnus resulted in the down-regulation of elevated expression level of *KISS-1* in PCOS animals. Vitagnus is rich in phytoestrogens and displays estrogenic-like activity (Liu et al., 2004a ▶). Allahtavakoli and his colleagues demonstrated that *Vitex agnus-castus*up-regulated the expression level of α-estrogen receptor gene in the hippocampus of ovariectomized rats (Allahtavakoli et al., 2015 ▶). Liu and colleagues isolated linoleic acid from the fruits of *Vitex agnus-castus*and showed that it simulates estrogen inducible genes via binding to estrogen receptors (Liu et al., 2004b ▶). Taken together, on the basis of our findings and previous studies, Vitagnus seems to be able to increase the estrogen level in PCOS animals due to its estrogen-like activity, and consequently, down-regulates the elevated level of *KISS-1* to normal state,which results in the regulation of the HPG axis (Clarkson et al., 2009 ▶; Gorkem et al., 2018 ▶). Women with PCOS present higher levels of serum kisspeptin, and the serum level of kisspeptin was suggested to be used as a marker of reproductive disorders and PCOS in women, so, the use of kisseptin antagonists can be considered in the treatment of PCOS (Umayal et al., 2019 ▶). The results of this study indicated that Vitagnus acts as a modifier of Kisspeptin pathway and could be considered an effective treatment forPCOS.

In conclusion, Vitagnus treatment of PCOS animals ledto modification of the *KISS-1* gene expression in the hypothalamus. As a result, Vitagnus extract acts at the highest level of the HPG axis by regulating the expression level of *KISS-1*, which can result in changes in the levels of the sex hormones and removal of PCOS symptoms. Benefits of Vitagnus in the treatment of PCOS, or other menstrual syndromes, can be explained by this mechanism of action. This result is noticeable due to identification of *Vitex agnus-castus*action at the hypothalamus level, which can be considered in clinical applications.
